# Redefining Lumpectomy Using a Modification of the “Sick Lobe” Hypothesis and Ductal Anatomy

**DOI:** 10.4061/2011/726384

**Published:** 2011-06-30

**Authors:** W. Dooley, J. Bong, J. Parker

**Affiliations:** Department of Surgery, The University of Oklahoma Breast Institute and Division of Surgical Oncology, The University of Oklahoma Health Sciences Center, Oklahoma, OK 73104, USA

## Abstract

*Objectives*. The “Sick Lobe” hypothesis states that breast cancers evolve from entire lobes or portions of lobes of the breast where initiation events have occurred early in development. The implication is that some cancers are isolated events and others are truly multi-focal but limited to single lobar-ductal units. *Methods*. This is a single surgeon retrospective review of early stage breast cancer lumpectomy patients treated from 1/2000 to 2/2005. Ductal endoscopy was used direct lumpectomy surgical margins by defining ductal anatomy and mapping proliferative changes within the sick lobe for complete excision. *Results*. Breast conservation surgery for stage 0–2 breast cancer with an attempt to perform endoscopy in association with therapeutic lumpectomy was performed in 554 patients (successful endoscopy in 465 cases). With an average followup of >5 years for the entire group, annual hazard rate for local failure in traditional lumpectomy without ductal mapping was 0.97%/yr. and for lumpectomy with ductal mapping and excision of entire sick lobe was 0.18%/yr. With endoscopy, 42% of patients were found to have extensive disease within their “sick lobe.” *Conclusions*. Targeting breast cancer lumpectomy using endoscopy and excision of regional associated proliferation seems associated with lower recurrence in this non-randomized series.

## 1. Introduction

The “Sick Lobe” hypothesis was proposed by Tibor Tot in 2005 [1]. His work was really a culmination of collecting relevant clinical and pathologic observations of the last century and a half. His first observations and predictions were based upon DCIS. The breast is defined as a single organ made of multiple lobes. Each lobe is identified by a single orifice on the nipple papilla connecting to branching tree of ducts and hundreds to thousands of individual lobules in the periphery. He proposed that for many cases of DCIS (especially extensive ones) the initiating events of carcinogenesis occurred perhaps as early as in the womb. Then throughout life as the lobe both grew and contracted from hormonal and other influences progression would occur at varying rates in different regions of the ductal tree. This led to the situation of apparent multifocality within the ductal tree and pathologic “skips” between DCIS patches. With further whole mount examination, extensive dissection of extensive intraductal component small invasive cancer cases, and multifocal invasive cancers, the findings support this theory [2–7]. Further molecular studies would seem to indicate that serious adverse genetic events are present throughout many ductal trees in what appears to be histologically normal tissue surrounding known cancers [8]. This is in direct conflict with older theories that the initial events all occurred at the terminal ductal lobular interface and spread pagetoid toward the nipple. The new theory then proposes that simultaneous or asynchronous malignant transformation occurs up and down ducts of the entire lobe and not as a result of migration. It also proposes that each lobe is relatively independent of the other so that multifocality within the lobar unit is common but multicentricity (simultaneous transformation in separate lobes) is rare. This last prediction has certainly held true in larger series of breast endoscopy where even widely separate tumors within a single breast are connected to the same duct system.

The problem comes in how do we turn this new theory into something useful to the operative surgeon trying to do the best job at breast conservation. The vast majority of early stage tumors we treat seem to involve relative small region of the ductal tree, and with current breast conservation surgery and radiation, few ipsilateral new tumors appear. If the whole ductal tree was genetically predisposed, then it would seem that we still should have more local failure events than we currently encounter. The Tibor Tot version of the sick lobe hypothesis would seem to indicate that a certain margin of histologically normal tissue may be inadequate to prevent recurrence. This clearly flies in face of work such as Mel Silverstein's Van Nuys index where margin seems paramount in predicting recurrence [9]. If followed to its full conclusion, Tot's theory would have us excising the entire lobe (ductal tree) associated with any new breast cancer. While feasible it does seem too extensive and because of complex branching technically difficult for the average breast cancer.

From 2000–2005, we performed a series of lumpectomies where the duct connecting the tumor with the nipple was attempted to be identified. When the duct identified, that duct was endoscoped to detect subclinical intraductal proliferative disease [10]. As has been previously reported this resulted in finding intraductal proliferative growths in 42% that extended beyond the image and clinical 1 cm planned excision margin. The ductal mapping revealed that often cancers were relatively distally located and associated proliferative disease seen endoscopically was limited to very short segments of adjoining ducts. In the case of EIC, ducts were extensively involved for long distances and had skips of greater than 2 cm commonly. Multifocal tumors arose in separate regions of the ductal tree at varying rates. Using the endoscopic ductal tree mapping of intraluminal disease, we elected to remove all intraductal proliferative disease associated with a known cancer independent of its histology (i.e., DCIS, ADH, ALH, DH, etc.). This was done back to at least a 1 cm length of normal duct in the nippleward direction. Once the duct was filled with tumor more distal branches could not be endoscoped so the resection was carried out in a pie-shaped wedge to the periphery to encompass those additional portions of the ductal tree.

New proposed Modified Sick Lobe Hypothesis-Surgical Practical Application.

We propose these changes/additions to the Tot “sick Lobe” hypothesis to address surgical planning.

1. Most breast cancers begin as isolated genetic events in a single-stem cell during expansion of the ducto-lobular tree.
2. The extent of the ductal tree involvement is reflective of the position of the stem cell where initiation events occurred. If occurring early and close to the nipple the tree will have extensive involvement distally manifested by large regions sharing abnormal genotype. If occurring relatively late in the development of the ductolobular tree, then regions derived from the initiating stem cell will be peripheral and limited within the tree. True pagetoid spread or spread by random migration up and down the ducts would be exceedingly rare.
3. Surgical lumpectomy should be best defined as the adequate removal of the potions of the genetic tree sharing the initiating genotype changes with the known breast cancer. This approach should decrease recurrence by eliminating metachrounous changes within the same ducto-lobular tree.

This hypothesis could then be tested by examining the local failure rates and patterns of local failures in the endoscopically directed lumpectomies as compared to those which were not.

## 2. Methods

This is a single surgeon review of patients treated at two institutions (Johns Hopkins and University of Oklahoma) from 1/2000 through 2/2005 with stages 0–2 breast cancer with breast conservation without any neoadjuvant chemo- or hormonal therapies.). All patients with prior periareolar resections, prior open surgical biopsies, or large hematomas associated with prior biopsy were not attempted. Otherwise this series includes all those with small tumors (<3 cm) requesting conservation as previously reported. Each patient had careful dekeratinization of the nipple in the operating room and then underwent centripetal breast massage using hand lotion. After the massage (which was also done after lymphazurin injection if sentinel node was also being performed), the retroareolar space was carefully compressed to identify all fluid producing orifices in the nipple papilla. The orifice yielding fluid closest to the position of the known cancer or yielding lymphazurin in the case or peritumoral injection was chosen for ductoscopy to identify the ductal connection to the tumor and associated proliferative disease. This was even done in cases of radiographic apparent multifocality or multicentricity. Ductal anatomy was drawn on the breast surface through the aid of transillumination in a darkened room. Regions of intraductal filling defects caused by epithelial proliferative growths were then marked as well. Lumpectomies were designed to remove known cancer and associated intraluminal growths as previously discussed and in keeping with the new modified sick lobe hypothesis.

## 3. Results

During this interval (2000–2005), there were 554 patients with early-stage breast cancer in which endoscopy was attempted (Table 1). Endoscopy was successfully completed and identified correctly the duct connecting with the tumor or immediate tumor region in 465 cases. In 16% of cases where no fluid producing duct was found or duct contained no abnormalities and did not connect to tumor region, lumpectomy was performed on the basis of clinical, radiographic, and ultrasonographic guidance as is standard for most breast surgeons.The average followup of these patients was 5.9 years for the endoscopically directed lumpectomies and 5.7 years for those not endoscopically directed (ranges 1.2–8 years). The annual hazard rate for local failure was 0.97% for traditional lumpectomy and 0.18% for those who had endoscopically directed excision of tumor and associated endoscopic lesions. This reaches statistical significance with the recurrence rate of 1.1% for endoscopically directed lumpectomy and 5.6% for traditional lumpectomy (*P* = 0.019; Chi Square, SPSS Ver.10 Chicago, IL). Diffuse involvement of the lobe was defined as extensive proliferative changes seen endoscopically greater than 1 cm from all clinical, radiographic, and ultrasonographic evidences of tumor. In some these involved the whole ductal tree but in most were subsegmental in distribution. At the time of these resections we had not anticipated the need to document volume and weight so we do not have consistently obtained information to compare these parameters in these cases. 

Since all patients were treated by NCCN guidelines or on clinical trials, there were no patients who did not receive radiation. All ER+ patients received hormonal therapy. Event rates of local recurrence remain low enough that no other treatment-related factors reach significance. Local ER-recurrences seem higher than ER-proportion in the entire group but this also fails significance.

## 4. Conclusions

The initial description of the “sick lobe” does fit many patients with extensive DCIS or multifocal DCIS and invasive disease [1]. Breast endoscopy strongly suggests that the clinically relevant genetic changes may be more widespread than initially radiographically appreciated changes but are still often subsegmental within an individual lobe [10, 11]. Much has been argued over the benefits of breast endoscopy since so many of the intraluminal defects are not invasive cancer or DCIS. Certainly the Cleveland Clinic experience directly shows that these additional lesions would not normally raise concern if found at the margin of traditionally performed lumpectomy [12]. As noted previously we took an alternate philosophical approach believing that regional proliferative changes present close to a cancer and not elsewhere were potentially sinister independent of the histopathologic changes they showed. Our prior report confirms that this assumption was associated with dramatic improvements in clear margins at first resection. This can rightfully be criticized in that these resections were bigger than those of nonendoscopically directed lumpectomy so of course margins would be better. However if we are truly affecting the natural history of breast cancer metachronous development within a sick lobe, we should see much fewer ipsilateral recurrences just as we have shown. Even though the absolute number of events is small, we are struck by the fact that contralateral breast cancers in these same patients seem to be occurring at almost identical rates as ipsilateral events in those patients with endoscopically directed proliferative disease included lumpectomies. Further in these patients the ipsilateral events seem randomly distributed and not clustered in the same quadrant as the initial primary as seen in nonendoscopically directed lumpectomy—either this series or others. Several other ductoscopy-directed lumpectomy series find regional proliferative disease in patterns identical to what we initially described in this series [11, 13–15].

Further we need to consider the classic idea of migration of DCIS up and down the ducto-lobular tree. If shed cells into the ductal fluid are totipotent, then we would expect that ductal installation of saline lavage or distending fluids associated with endoscopy would likely result in spreading of disease. This should be manifested by increased local failure events in the ipsilateral breast. Our data does not find this. Similarly researchers using ductal lavage in cancer patients did not see increased local failures. One could argue that such events are masked by the use of radiation for breast conservation in these patients and that it is a valid possibility for suppression of local recurrences. As more surgical investigators become facile with ductal endoscopic mapping, a clinical trial of endoscopically mapped lumpectomy could test whether radiation therapy is still needed if we actually can do an anatomically defined lumpectomy. We therefore find that our follow-up data on ductoscopically directed lumpectomy supports a new version of the “sick lobe” hypothesis that directly addresses how surgeons think and procede to surgical planning around the time of lumpectomy. We view that breast cancer is a lobar disease isolated to the section of the lobe where the initiation events occurred and all subsequent outgrowth from those stem cells. With this change, breast cancer may be isolated to a distal branch of the ducto-lobular tree when initiation events occurred late in lobar growth. These would be the common tumors with little surrounding proliferative disease and well treated by our current techniques. If these could be better defined by the genetic mapping of changes in ducts in their region, simple excision or ablation of all genetically abnormal epithelium of the duct without radiation might be adequate for their control. Some tumors develop from earlier initiation events but still isolated to larger segmental regions within the ducto-lobular tree. Here excisions would need to either be wider or have associated radiation to eliminate the potential progression of genetically altered proliferative cells. The extent of this abnormal proliferation within the segment would determine also how much irradiation if any needed (i.e., no radiation, partial breast irradiation, or whole breast irradiation). Finally as in the classic Tibor Tot description, the entire lobe is occasionally involved because the initiation events occurred in some of the first stem cells of the lobe early in its development. Here either complete lobe excision or whole breast treatment may be required. We agree with Tibor Tot that true multicentricity of cancers developing in differing lobes of the same breast should be extremely rare [5]. In fact we would propose given our preliminary data that it should be no more common than synchronous or metachronous contralateral breast cancers.

This new hypothesis then raises important clinically relevant questions. Can a better lumpectomy be performed when guided by ductal anatomy and the plan to completely excise proliferative changes sharing genetic signatures with the primary tumor? Are our current tools adequate to embark on an exploration of this hypothesis? If not, what needs to be developed? Most submillimeter endoscopy systems currently have limited biopsy capabilities. Can these be changed or improved so that molecular mapping of the ductal tree can be efficiently performed and a more biologically appropriate surgical approach be taken to lumpectomy? If an anatomic molecular mapped lumpectomy is feasible, can we begin to consider elimination of radiation therapy in early-stage breast cancer without LVI as an appropriate arm in a clinical trial? As our molecular understanding and genotyping of breast cancers become more commonplace, we as surgeons need not to consider that these techniques are only ways to choose better adjuvant therapies but reassess our technical approaches to breast cancer. We are still doing the lumpectomy technique of Bilroth in the mid 1800's with only more careful attention to histopathologic margin. It is time for us to consider applying new and evolving breast cancer biology information to improving the technical aspects of local therapy.

Our data suggests that this new approach to lumpectomy may be valid but can be criticized since increasing volume of resection in certain cases would naturally be expected to decrease recurrence. The Tot theory and our guidelines for application to lumpectomy represent a major deviation from traditional breast cancer biology theories. We encourage others to consider these theoretical proposals and test them against their own observations. The evolution of this new sick lobe hypothesis and the ability to do real time ductal mapping via ductoscopy should strongly motivate surgical innovators to perform multicenter prospective randomized trials to test the validity of this new theory and approach. If accurate, this approach would fundamentally change local therapy for breast cancer.

## Figures and Tables

**Table 1:** Case distribution and results.

Age	Mean	
Range 32–89	57	
Tumor stage		
DCSIS	155	28%
Stage 1 or 2	399	72%
# of successful endoscopies	465	84%
# BC with additional lesions		42.1%
If endoscopy not successful		
margin +		19.1%
If endoscopy successful		
margin +		4.7%
If endoscopy successful		
nipple ward margin +		0.36%
Annual hazard rate for L/R recurrence		
with endoscopic guidance		0.18%
Annual hazard rate for L/R recurrence		
without endoscopic guidance		0.97%
